# Social connection and physical health outcomes among long-term care home residents: a scoping review

**DOI:** 10.1186/s12877-021-02638-4

**Published:** 2021-12-18

**Authors:** Kaitlyn Lem, Katherine S. McGilton, Katelynn Aelick, Andrea Iaboni, Jessica Babineau, Debbie Hewitt Colborne, Cathleen Edwards, Monica Bretzlaff, Dee Lender, Josie-Lee Gibson, Jennifer Bethell

**Affiliations:** 1 Faculty of Arts & Sciences, University of Toronto, Toronto, ON Canada; 2 KITE Research Institute, Toronto Rehabilitation Institute – University Health Network, Toronto, ON Canada; 3 Lawrence S. Bloomberg Faculty of Nursing, University of Toronto, Toronto, ON Canada; 4 Behavioural Supports Ontario Provincial Coordinating Office, North Bay Regional Health Centre, North Bay, ON Canada; 5 Department of Psychiatry, University of Toronto, Toronto, ON Canada; 6 Library and Information Services, University Health Network, Toronto, ON Canada; 7 Family Councils Ontario, Toronto, ON Canada; 8 Ontario Association of Residents’ Councils, Newmarket, ON Canada; 9 Institute of Health Policy, Management and Evaluation, University of Toronto, Toronto, ON Canada

**Keywords:** Scoping review, Long-term care, Older adults, Nursing home, Physical health, Social connection

## Abstract

**Background:**

Social connection is recognized as an important determinant of health and well-being. The negative health impacts of poor social connection have been reported in research in older adults, however, less is known about the health impacts for those living in long-term care (LTC) homes. This review seeks to identify and summarize existing research to address the question: what is known from the literature about the association between social connection and physical health outcomes for people living in LTC homes?

**Methods:**

A scoping review guided by the Arksey & O’Malley framework was conducted. Articles were included if they examined the association between social connection and a physical health outcome in a population of LTC residents.

**Results:**

Thirty-four studies were included in this review. The most commonly studied aspects of social connection were social engagement (*n* = 14; 41%) and social support (*n* = 10; 29%). A range of physical health outcomes were assessed, including mortality, self-rated health, sleep, fatigue, nutrition, hydration, stress, frailty and others. Findings generally support the positive impact of social connection for physical health among LTC residents. However, most of the studies were cross-sectional (*n* = 21; 62%) and, of the eleven cohort studies, most (*n* = 8; 73%) assessed mortality as the outcome. 47% (*n* = 16) were published from 2015 onwards.

**Conclusions:**

Research has reported positive associations between social connection and a range of physical health outcomes among LTC residents. These findings suggest an important role for social connection in promoting physical health. However, further research is needed to consider the influence of different aspects of social connection over time and in different populations within LTC homes as well as the mechanisms underlying the relationship with health.

**Supplementary Information:**

The online version contains supplementary material available at 10.1186/s12877-021-02638-4.

## Background

Social connection (including social networks, social engagement, social support, and loneliness) is recognized as a key determinant of health and well-being [1, 2]. Systematic reviews and meta-analyses suggest social connection influences not only mental health, but also physical health outcomes, including mortality [3, 4], coronary heart disease, and stroke [5]. A 2015 meta-analysis found that low social integration and low social support, two aspects of social connection, had a greater impact on mortality than smoking, body mass index (BMI), physical activity, and alcohol consumption [3]. The mechanisms underlying these associations have been postulated to include immune system function [6], stress regulation [7], and health behaviours (e.g., diet and exercise) [8]. Loneliness and social isolation, concepts reflecting poor quality or quantity of social connection, have also been posited to create barriers to healthy behaviour engagement and treatment plan adherence [9, 10].

Social isolation and loneliness are widespread in LTC homes [11, 12], however, relatively little is known about the health impacts in this setting [12–14]. Given the characteristics of LTC homes and their residents [15, 16], research is needed to specifically address issues of social connection in this population [12, 14]. For example, LTC homes have an integral role in enabling social connection for residents, such as through the home environment (e.g. shared living spaces, planned social and recreational activities) or promoting ongoing support when separated from family [17]. However, amid the current COVID-19 pandemic, LTC homes have restricted visitors (including family and friends) and group activities [18, 19] as a means of infection-control, which has had devastating impacts on social connection for residents [18, 20].

To our knowledge, there are no published reviews on the association between social connection and physical health among LTC home residents. To address this gap, the objective of this scoping review is to identify and summarize the existing research to address the question: what is known from the literature about the association between social connection and physical health outcomes for people living in LTC homes? We chose scoping review methodology to answer a broad research question by assessing published literature in which we anticipated a heterogeneous list of exposures and health outcomes, then to identify and report knowledge gaps [21–23].

## Methods

This scoping review is a sub-analysis of a larger scoping review, initiated prior to the COVID-19 pandemic, to address a broad set of research questions on social connection among LTC home residents. A protocol for the larger scoping review has been published [14], and is briefly summarized below along with specifications for the present study, which represents the second publication stemming from the results of the larger scoping review. The results are being presented in multiple papers as a response to the volume of research studies located in our search as well as the immediate need to address knowledge gaps created by COVID-19. More specifically, the first publication was a direct response to the COVID-19 pandemic and research funding from the Canadian Institutes of Health Research; it focused on summarizing research on mental health impacts of social connection and potential strategies during COVID-19, which were knowledge gaps identified by our partner organisations (see section Consulting with stakeholders and knowledge users) [24]. A third publication is also in preparation, summarizing research reporting associations between LTC home- and community-level characteristics and resident social connection, which was also identified as an important knowledge gap.

The review followed the six-stage scoping review framework as developed by Arksey & O’Malley [25] and Levac et al. [22] and report results in accordance with the PRISMA Extension for Scoping Reviews [23].

### Searching for relevant literature

A literature search was conducted to identify relevant published journal articles that reported a quantitative measure of social connection among residents of LTC homes. Inclusion criteria specified settings described as LTC homes, nursing homes, or care homes. These terms were selected to reflect terminology differences between countries but also to reflect overlap and international consensus on the definition of nursing home [11]. In this paper, they are hereafter collectively referred to as LTC homes.

The following concepts were included as aspects of social connection: [26] *social networks*, the size and nature of the social network structure as well as characteristics of the social ties, and acknowledge that these networks provide opportunities for social support and social engagement [27]; *social engagement*, taking part in real-life activities with others [27, 28] as well as *social disengagement* [29]*; social support*, instrumental, emotional, appraisal, and informational help available [27] and *social isolation*, a lack of personal relationships [30]; *social capital*, the features of relationships that facilitate mutual benefits, such as interpersonal trust, reciprocity, and mutual aid [31]. The subjective experiences of *loneliness* [32], *social connectedness* [33], and *perceived isolation* [34] were also included.

For the current scoping review sub-analysis, studies reporting physical health outcomes were eligible for inclusion if they quantified any phenomenon that impacts the bodies’ function, form, or structure [35], including self-assessed health [36, 37]. Mental health outcomes were excluded as they were addressed in a separate publication [24]. Quality of life and well-being outcomes were also excluded [38]. Given the practical considerations of analyzing the volume of research anticipated in the larger scoping review and our original intent to map gaps in observational and interventional research, we did not include qualitative studies in this scoping review [14]. Although both observational and intervention studies were eligible for inclusion in the larger scoping review, we did not include studies that target social connection without also including a quantitative measure of social connection; for example, studies of interventions that address social connection but report only physical health outcomes would not be included in this review. See Table 1 for a summary of inclusion criteria.

**Table 1:** Summary of the scoping review inclusion criteria

Inclusion criteria	
Element	Inclusion details
Population	LTC home residents
Exposure	Social connection (including social networks, social engagement, social disengagement, social support, social isolation, social capital, loneliness, and social connectedness)
Comparator	Any
Outcome	Physical health

The original search strategy was conducted by an information specialist who searched in MEDLINE(R) ALL (in Ovid, including Epub Ahead of Print, In-Process & Other Non-Indexed Citations, Ovid MEDLINE(R) Daily) and then translated into CINAHL (EBSCO), APA PsycINFO (Ovid), Scopus, Sociological Abstracts (Proquest), Embase and Embase Classic (Ovid), Emcare Nursing (Ovid) and AgeLine (EBSCO). Initial searches were conducted from the databases’ inception through to the date the search was executed (July 2019) and limited to the English language. The search strategies were updated with additions to acknowledge changes in the MeSH (Medical Subject Headings) indexing terms and the searches were executed again in January 2021 (see Additional file 1). Covidence (www.covidence.org) and Endnote were used for the review process and deduplication of database results [39].

### Selecting studies

**Fig. 1:**
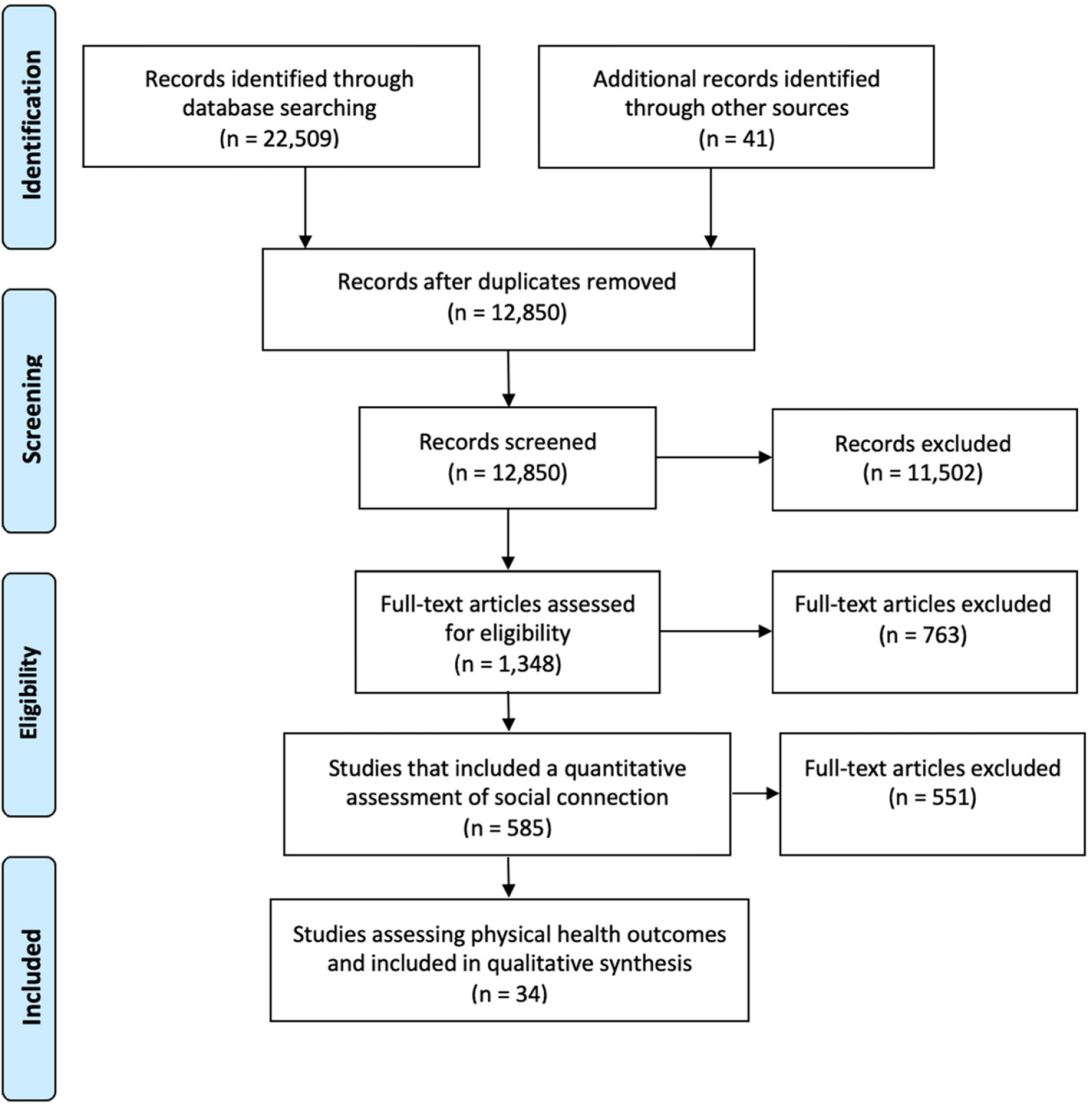
PRISMA Diagram

As part of the larger scoping review, to identify studies that reported a quantitative measure of social connection among residents of LTC homes, two reviewers independently screened article titles and abstracts and then full-text articles. Two reviewers also independently reviewed these articles to identify studies that reported associations between social connection and physical health outcomes (See Fig. 1 PRISMA flow diagram). In the case of disagreement, the reviewers discussed and resolved conflicts.

### Charting the data

A data abstraction form was created using Google forms and Google sheets to facilitate data extraction. Data charting was done independently and in duplicate. Given the nature of this review, no quality assessments of the studies were taken. Data were abstracted on article characteristics (e.g., country), objective, study design, setting (e.g., described as a nursing home or LTC home), sample (e.g., number of residents, mean age, sex distribution, inclusion/exclusion criteria related to cognition), measure of social connection, physical health outcome and a summary of study findings linking social connection and physical health outcomes.

### Collating, summarizing, and reporting the results

Study characteristics were summarized with descriptive statistics, as well as a narrative synthesis. For the narrative synthesis, we took a descriptive-analytical approach [25]. The study team postulated a theoretical model for the mechanisms underlying the association between social connection and physical health outcomes [6–10]. Study characteristics were then organized to describe patterns and explore potential factors contributing to direction and size of effect. The first author reported consolidated results back to the study team who reviewed the results, suggested refinements, and provided insights on the findings.

### Consulting with stakeholders and knowledge users

The initial protocol [14] describes opportunities to present to and engage with LTC home residents, families, and staff, however, COVID-19 made in-person engagements impossible. Thus, for this review, we worked with partners from organizations who represent LTC knowledge users: Behavioural Supports Ontario Provincial Coordinating Office, Ontario Association of Residents’ Councils, and Family Councils Ontario. These members of our study team were involved in priority-setting (defining the review questions), analyzing data, interpreting and contextualizing the results, and coauthoring the current review.

## Results

### Study characteristics

As part of the larger scoping review [14], the search strategy yielded 22,509 titles, which reduced to 12,850 after deduplication. The list was distilled to 585 papers that quantified social connection in LTC residents, from which 34 papers were identified for the current analysis (see Fig. 1).

Characteristics of included studies are described in Additional file 2 and summarized in Tables 2, 3 and 4. Over two thirds (*n* = 23; 68%) of the studies were published in or after 2010 and just under half (*n* = 16; 47%) published in 2015 or later. The largest proportion of studies were from North America (*n* = 13; 38%), mostly the United States (*n* = 11; 32%). Just under two-thirds (*n* = 21; 62%) of the studies were cross-sectional and most of the remainder were cohort studies (*n* = 11; 32%). Among the eleven cohort studies, the majority (*n* = 8; 73%) assessed mortality as the outcome. Most of the studies described the setting as nursing homes (*n* = 27; 79%). The number of homes included in the studies ranged from 1 to 653, with a median of 6. The number of LTC residents included in the studies ranged from 40 to 30,055, with a median of 503.5. Mean resident age ranged from 68 to 87, with a median of 83.3. The proportion of females included in the studies ranged from 0 to 87% with a median of 71%.

**Table 2:** Characteristics of studies included in scoping review

Study characteristics	
Total articles n (%)	34 (100)
Year n (%)	
1995–1999	2 (5.9)
2000–2004	5 (14.7)
2005–2009	4 (11.8)
2010–2014	7 (20.6)
2015+	16 (47.1)
Country n (%)	
United States	11 (32.4)
Taiwan	4 (11.8)
China	4 (11.8)
Iceland	2 (5.9)
Portugal	2 (5.9)
Spain	2 (5.9)
Italy	2 (5.9)
Canada	2 (5.9)
Other^a^	12 (33)
Multiple^b^	1 (2.9)
Study design n (%)	
Cross-sectional	21 (61.8)
Cohort	11 (32.4)
Ecologic	1 (2.9)
Not Stated	1 (2.9)
Study setting	
Nursing home	27 (79.4)
Long-term care	7 (20.6)
Number of institutions	
# of articles reporting n (%)	29 (85.3)
Range	1–653
Median	6
Interquartile Range (IQR)	42
Number of residents (participants)	
# of articles reporting n (%)	34 (100.0)
Range	40–30,055
Median	503.5
IQR	1020.5
Mean age of residents	
# of articles reporting n (%)	24 (70.6)
Range	68–87
Median	83.3
IQR	3.5
Percentage of females	
# of articles reporting n (%)	32 (94.1)
Range	0–87
Median	70.6
IQR	14.4

^a^Other countries included Denmark, Lebanon, Iran, Hong Kong, Czech Republic, Finland, England, France, Germany, the Netherlands, Norway, and Israel

^b^Total greater than n=34 (100%) with one international study conducted in 8 countries

# number

**Table 3:** Characteristics of social connection measures used in studies included in scoping review

Social connection exposures characteristics	
Total articles n (%)	34 (100)
Aspect of social connection	
Social engagement	14 (41.2)
Social support	10 (29.4)
Loneliness	6 (17.6)
Social network	2 (5.9)
Other^a^	6 (17.6)
Instrument/Method	
Minimum Data Set	12 (35.3)
UCLA Loneliness Scale	3 (8.8)
Leisure Social Support Scale	2 (5.9)
Other^b^	18 (52.9)

^a^Other measures of social connection included social integration, social interaction, social participation, social relationships, social involvement, and social isolation

^b^Other instruments/methods used to assess social connection include the Duke Older Americans Resources and Services Procedures, the Hebrew Home Social Network Rating Scale, a survey, interviews, a Modified Inventory of Socially Supportive Behaviours, behavioural observations, the Social Engagement Scale, the VAUX Social Support Questionnaire, a single question, the interRAI-LCTF, family visits, the Cohen-Mansfield measure of social network (1992), the Multidimensional Scale of Perceived Social Support, a single-item question, the Loucks Social Network Score, the De Jong Gierveld Scale, the Social Provisions Scale, and the Personal Resource Questionnaire

**Table 4:** Characteristics of physical health outcomes used in studies included in scoping review

Physical health outcomes characteristics	
Total articles n (%)	34 (100)
Physical health outcome categories	
Mortality	8 (23.5)
Self-rated health	6 (17.6)
Sleep and fatigue	5 (14.7)
Nutrition and hydration	5 (14.7)
Stress	2 (5.9)
Frailty	2 (5.9)
Other^a^	6 (17.6)

^a^Other measures of physical health included functional decline, successful aging, pain, self-feeding dependence over time, MRSA carriage, and multiple (including bladder or bowel incontinence, urinary tract infections, faecel impaction, little or no activity, bedfast residents, stage 1–4 ulcers, and falls)

### Social connection and physical health outcomes in LTC home residents

The most common aspects of social connection assessed were social engagement, social support, and loneliness (see Table 3). The physical health outcomes were categorized as mortality, self-rated health, sleep and fatigue, nutrition and hydration, stress, frailty and other (see Table 4).

#### Mortality

Eight studies assessed the association between social connection and mortality. Seven studies reported higher social connection (social engagement or support) were associated with reduced risk of mortality [40–46]. The eighth study reported an unadjusted association between social network quality, but not size, with results suggesting this relationship may depend on sex and cognitive impairment, but the associations were not statistically significant in multivariable models [47].

#### Self-rated health

Six studies assessed the association between social connection and self-rated health. One study found that loneliness was associated with poor health among women but not men [48]. Another found that, when considering social engagement within the home, outside the home (family and friends), and relatives within the facility, only infrequent contacts within the LTC home were associated with worse health [49]. Two studies used path analysis to consider the role of social support in predicting self-rated health status [50, 51]. Another two studies found no statistically significant association between social connection and self-rated health [52, 53].

#### Sleep and fatigue

Five studies assessed the association between social connection and sleep and fatigue. Two studies reported social support was inversely associated with sleep problems [54, 55] whereas another found social network (but not social support) was positively associated with fatigue [56]. Two studies reported social engagement was associated with less daytime sleep [57, 58].

#### Nutrition and hydration

Five studies assessed the association between social connection and nutrition and hydration. Results from the studies varied. One study found the association between loneliness and risk of malnutrition or being malnourished was no longer statistically significant after adjusting for appetite, eating difficulties due to oral health problems, symptoms of depression and functional status [59]. Similarly, another study found the association between social engagement and energy intake was no longer statistically significant after adjusting for eating challenges [60]. One study found a higher prevalence of low social engagement among underweight residents (BMI < 20) [61]. Another study found that differences in social isolation and loneliness scores according to nutrition status groups (i.e. malnutrition, at risk of malnutrition, and satisfactory nutritional status) were not statistically significant [62]. One study reported low social engagement was associated with dehydration but not weight loss [63]. Another study found low social engagement predicted increased self-feeding dependence [64].

#### Stress

Two studies assessed the association between social support and stress; in the first, social support was associated with acute (but not chronic) stress [65] and, in the second, emotional support was inversely associated with stress but instrumental support was not [66].

#### Frailty

Two studies assessed the association between loneliness and frailty; one study reported loneliness was associated with frailty [67] and another study suggested the relationship may be mediated by activity engagement [68].

#### Other physical health outcomes

Six studies assessed a range of other physical health outcomes. An ecological study found an association between social engagement and reduced MRSA transmission [69]. Another study tested the association between social engagement and a range of health outcomes and quality indicators (including bladder and bowel health, bedfast residents, pressure ulcers, and falls); low social engagement was associated with reduced risk of an indwelling catheter but increased risk of faecal impaction and bedfast state [63]. Another study found social engagement was associated with functional decline, but only in unadjusted analysis [70]. Social support was not associated with successful aging [71] or pain [72] and social engagement was not associated with urinary incontinence [73].

## Discussion

This scoping review of published research identified 34 studies that assessed the association between social connection and physical health in LTC residents. The studies reported a range of physical health outcomes, most commonly mortality, self-rated health, sleep, fatigue, nutrition, hydration, stress, and frailty. While the association between social and physical health has been studied for decades [74], research specific to LTC homes has received less attention [12]. To our knowledge, this review is the first to highlight research on the physical health outcomes of social connection in this population.

When considered together, the studies included in this review highlighted several knowledge gaps. First, aside from the studies of mortality, almost all (*n* = 21; 62%) the studies were cross-sectional, making temporal relationships impossible to determine for some outcomes; while it is possible that social connection impacts health, the impact of some of these same measures of health status (e.g., pain [75] and sleep [76]) on social connection have also been reported. Second, while the cohort studies identified in this review suggested consistent evidence of a potential protective effect for social connection on mortality among LTC residents, many studies did not test the potential mechanisms underlying the association between social connection. In particular, there was a lack of analysis on the potential biological [6, 7] and behavioural [8, 9] underpinnings that may explain the physical health outcomes associated with the multiple aspects of social connection being studied. The results of the current review, taken in context with a recent review on social connection and mental health outcomes in this population [24], suggest mechanisms consistent with previously proposed models [4, 77], but which highlight specific psychological factors (e.g., stress, depression) and lifestyle (e.g., nutrition, sleep) which warrant further research in this population. For example, with respect to the latter, addressing social connection as a means to improving nutritional status – either through individual-delivered interventions [78] or addressing aspects of the LTC home mealtime environment [79] – has been investigated. Third, few studies stratified results to compare populations within LTC homes (e.g., by sex or gender [47–49, 59], cognition [

While we acknowledge the policy and clinical practice implications from this scoping review are limited [21], our study identified a body of research on the health impacts of social connection that extends specifically to LTC residents. Set against the impacts of the COVID-19 pandemic - and extending beyond it - our findings underscore how social connection is essential in LTC homes [85], the vital role of essential/designated care partners (i.e., family and friends) [86, 87] and the importance of integrating LTC residents’ social connection as a predictor of physical and mental health [24] and a measure of quality of life [88] and care [89, 90].

## Limitations

To our knowledge, this is the first scoping review of research assessing the association between social connection and physical health specifically among LTC residents. We have highlighted knowledge gaps and identified opportunities for future research, however, the results must be interpreted in context with limitations. First, this is a scoping review [22, 23, 25] and the objective and methods did not include an examination of the quality of evidence; the implications of the results are primarily to guide future research in this area. Second, only English language studies were included and so the geographical distribution of studies may reflect this eligibility criterion and limit the generalizability of the findings [91, 92]. Third, inconsistent use of terminology related to the main concepts of the initial literature search (i.e., LTC homes [11] and aspects of social connection [31]) may have contributed to some research papers being omitted from the review. Fourth, the literature search only included intervention studies which reported social connection outcomes [24], thereby precluding interventions studies from this review.

## Conclusions

This study identified and summarized published research testing the association between social connection and physical health for people living in LTC homes. While a diverse range of outcomes were assessed, findings generally supported the positive association between social connection and physical health. Still, further research is needed to consider the influence of social connection on health trajectories, compare populations within LTC homes, integrate multiple aspects of social connection, and assess the distinct mechanisms through which these aspects of social connection might influence health in this population.

## Supplementary Information

**Additional file 1.** Search Strategy.

**Additional file 2.** Study Characteristics.

## Data Availability

The datasets used and/or analyzed during the current study are available from the corresponding author on reasonable request.

## Abbreviations

LTC: Long-Term Care
BMI: Body Mass Index
MDS: Minimum Data Set
MRSA: Methicillin-resistant *Staphylococcus Aureus*
