# Supplementary material for: Social connection and physical health outcomes among long-term care home residents: a scoping review
Source: BMC Geriatr. 2021 Dec 18;21:722. doi: 10.1186/s12877-021-02638-4 (PMC8683818; doi:10.1186/s12877-021-02638-4)
Supplement: Supplementary file 1 — Additional file 1. Search Strategy. [file 12877_2021_2638_MOESM1_ESM.docx]

**Additional File 1: Search Strategy**

**Title:** Social connection and physical health outcomes among long-term care home residents: A scoping review

**Authors:** Kaitlyn Lem BSc, Katherine S. McGilton RN, PhD, FAAN, FCAHS, Katelynn Aelick MSc, Andrea Iaboni MD, DPhil, Jessica Babineau MLIS, Debbie Hewitt Colborne RN, MScN, Cathleen Edwards MA, Monica Bretzlaff BA, TRS, Josie-Lee Gibson, Dee Lender, and Jennifer Bethell PhD

**Search Narrative**

This strategy was first developed in Medline, and was then translated to other databases. It uses a number of concepts:

Concept A: lines 1 to 38 = Social engagement, social support, social capital, loneliness or social isolation

Concept B: lines 39 to 58 = Long term care, or nursing homes, homes for the aged

The search conducted is: (A AND B)

This strategy was used in all searched databases. Searches were also limited to adult human populations, and English language when possible.

In other databases, additional limits and search fields have been used when applicable such as to exclude conference proceedings.

**Medline (Ovid) Search Strategy**

1 Interpersonal Relations/

2 exp social support/

3 Social Participation/

4 exp Social Isolation/

5 Social inclusion/

6 Social Interaction/

7 Social Network Analysis/

8 Loneliness/

9 Social Behavior/

10 ((social or socially) adj3 (engaged or engagement)).tw,kf.

11 ((social or socially) adj3 (disengaged or disengagement)).tw,kf.

12 ((social or socially) adj3 (connect* or disconnect*)).tw,kf.

13 Sociali?ation.tw,kf.

14 Sociali??.tw,kf.

15 sociali?ing.tw,kf.

16 Interpersonal relation?.tw,kf.

17 (social adj3 behavio?r*).tw,kf.

18 (social adj3 capital?).tw,kf.

19 ((social or socially) adj3 support??).tw,kf.

20 (social adj3 relationship?).tw,kf.

21 ((social or socially) adj3 participat*).tw,kf.

22 (social adj3 network?).tw,kf.

23 (social adj3 interaction*).tw,kf.

24 (personal adj3 network?).tw,kf.

25 friendship?.tw,kf.

26 ((social or socially) adj3 integrat*).tw,kf.

27 (social adj3 relation?).tw,kf.

28 (social adj3 contact).tw,kf.

29 ((social or socially) adj3 embedded*).tw,kf.

30 lonel*.tw,kf.

31 ((social or socially) adj3 isolat*).tw,kf.

32 (emotional* adj3 isolat*).tw,kf.

33 ((perceive* or percept*) adj3 isolat*).tw,kf.

34 ((social* or socially) adj3 alienat*).tw,kf.

35 aloneness.tw,kf.

36 solitud*.tw,kf.

37 Unwanted alone.tw,kf.

38 or/1-37

39 Nursing Homes/

40 Residential Facilities/

41 Homes for the Aged/

42 Long-Term Care/

43 "long-term care".tw,kf.

44 "longterm care".tw,kf.

45 LTC.tw,kf.

46 ((home? or facility or facilities) adj2 (retirement or assisted living or residential care or extended care)).tw,kf.

47 home? for the aged.tw,kf.

48 home? for the elderly.tw,kf.

49 old age home?.tw,kf.

50 ("old age facility" or "old age facilities").tw,kf.

51 (nursing adj3 (facility or facilities or residence? or center? or centre?)).tw,kf.

52 nursing home?.tw,kf.

53 (residence? adj2 ('assisted living' or convalescen* or retire???? or 'long stay' or longstay or 'long term')).tw,kf.

54 ((facility or facilities) adj2 ('assisted living' or convalescen* or retire???? or resident* or 'long stay' or longstay or 'long term')).tw,kf.

55 (home? adj2 ('assisted living' or convalescen* or retire???? or 'long stay' or longstay or 'long term')).tw,kf.

56 "care home?".tw,kf.

57 (housing adj3 (retirement or old age or senior* or elderly)).tw,kf.

58 or/39-57

59 38 and 58

60 59 not ((exp infant/ or exp child/) not exp adult/)

61 60 not (exp animals/ not exp humans/)

62 limit 61 to english language
